# Loneliness and social isolation are associated with an increased risk of glaucoma: a UK Biobank cohort study

**DOI:** 10.1186/s12889-024-19649-6

**Published:** 2024-08-05

**Authors:** Xinyu Zhu, Bo Li, Xinyu Zhang, Yujin Jiang, Yikeng Huang, Chenxin Li, Zhi Zheng, Yili Zhang, Bei Zhu, Shuzhi Zhao

**Affiliations:** 1 Department of Ophthalmology, Shanghai General Hospital, Shanghai Jiao Tong University School of Medicine, Shanghai, 200080 China; 2 National Clinical Research Center for Eye Diseases, Shanghai Key Laboratory of Ocular Fundus Diseases, Shanghai Engineering Center for Visual Science and Photomedicine, Shanghai Engineering Center for Precise Diagnosis and Treatment of Eye Diseases, Shanghai, 200080 China; 3 Department of Ophthalmology, the Fourth Affiliated Hospital of Soochow University, Suzhou, Jiangsu Province 215123 China; 4 Jiuting Community Health Service Center, Songjiang District, Shanghai, 201615 China; 5 Ningde Municipal Hospital, Ningde Normal University, Ningde, Fujian Province 352100 China; 6 Fujian Medical University, Fuzhou, Fujian Province 350122 China

**Keywords:** Association, Glaucoma, Loneliness, Risk, Social isolation

## Abstract

**Background:**

Loneliness and social isolation have been found to be associated with various health-related outcomes. Our study aimed to evaluate the association of loneliness and social isolation with the risk of glaucoma.

**Methods:**

A total of 373,330 participants from the UK Biobank without glaucoma at recruitment were included in this study. Self-reported questionnaires were used to define loneliness and social isolation. Incident glaucoma events were identified by hospital inpatient admissions and self-reported data. COX proportional hazards models adjusted for sociodemographic, lifestyle, and health-related factors were used to estimate hazard ratios (HRs) and 95% CIs.

**Results:**

During a median follow-up of 13.1 (interquartile range: 12.3–13.9) years, 6,489 participants developed glaucoma. After adjusting for confounding factors, loneliness (yes vs. no: adjusted HR: 1.16; 95% CI: 1.04–1.30; *P* = 0.009) and social isolation (yes vs. no: adjusted HR: 1.08; 95% CI: 1.01–1.16; *P* = 0.033) were associated with an increased risk of glaucoma.

**Conclusions:**

In this population-based prospective cohort study, loneliness and social isolation were associated with a higher risk of glaucoma.

**Supplementary Information:**

The online version contains supplementary material available at 10.1186/s12889-024-19649-6.

## Background

Glaucoma, characterized by progressive optic neuropathy and visual field loss, remains among the leading causes of irreversible blindness worldwide [1, 2]. It is predicted that 111.8 million individuals will have glaucoma by 2040, putting a strain on society and individuals [3]. Complex interplay of genetic, systemic, and environmental factors contributes to the development of glaucoma [1, 4]. However, the specific pathogenesis of glaucoma warrants further studies. Therefore, it is crucial to identify potentially modifiable risk factors to reduce the onset or improve the prognosis of this disease.

Loneliness and social isolation are established social problems which have a negative impact on both mental and physical health [5]. Loneliness is a subjective feeling caused by a disparity between a person’s real and desired degree of social connections, whereas social isolation is an objective experience of a lack of social activities [6, 7]. Emerging evidence has suggested that loneliness and social isolation are associated with a higher risk of various health-related outcomes, such as dementia [8], cardiovascular diseases [9–11], and hospital-treated infections [12]. Nevertheless, little is known concerning their associations with the incidence of glaucoma.

Some plausible mechanisms may connect loneliness and social isolation with the risk of glaucoma. It is reported that loneliness and social isolation may impact health through biological changes such as increased cortisol secretion and elevated levels of inflammation [13, 14]. These changes may lead to elevated intraocular pressure (IOP) and accelerated death of retinal ganglion cells (RGCs), thereby promoting the onset of glaucoma [15, 16]. From a public health perspective, exploring the link between loneliness and social isolation with the risk of glaucoma may help to identify at-risk populations for early prevention and management of glaucoma.

Hence, we aimed to prospectively evaluate the association of loneliness and social isolation with the risk of glaucoma among participants from the UK biobank.

## Methods

### Study design and population

The UK Biobank is a large population-based cohort study of more than 500,000 participants aged between 40 and 69 years. Participants were recruited from 22 assessment centers across the United Kingdom during 2006 and 2010. All participants provided written informed consent, and the study gained ethics approval from the North West Multicenter Research Ethics Committee (REC reference NO.11/NW/0382). Details of the cohort study have been discussed previously [17].

**Fig. 1:**
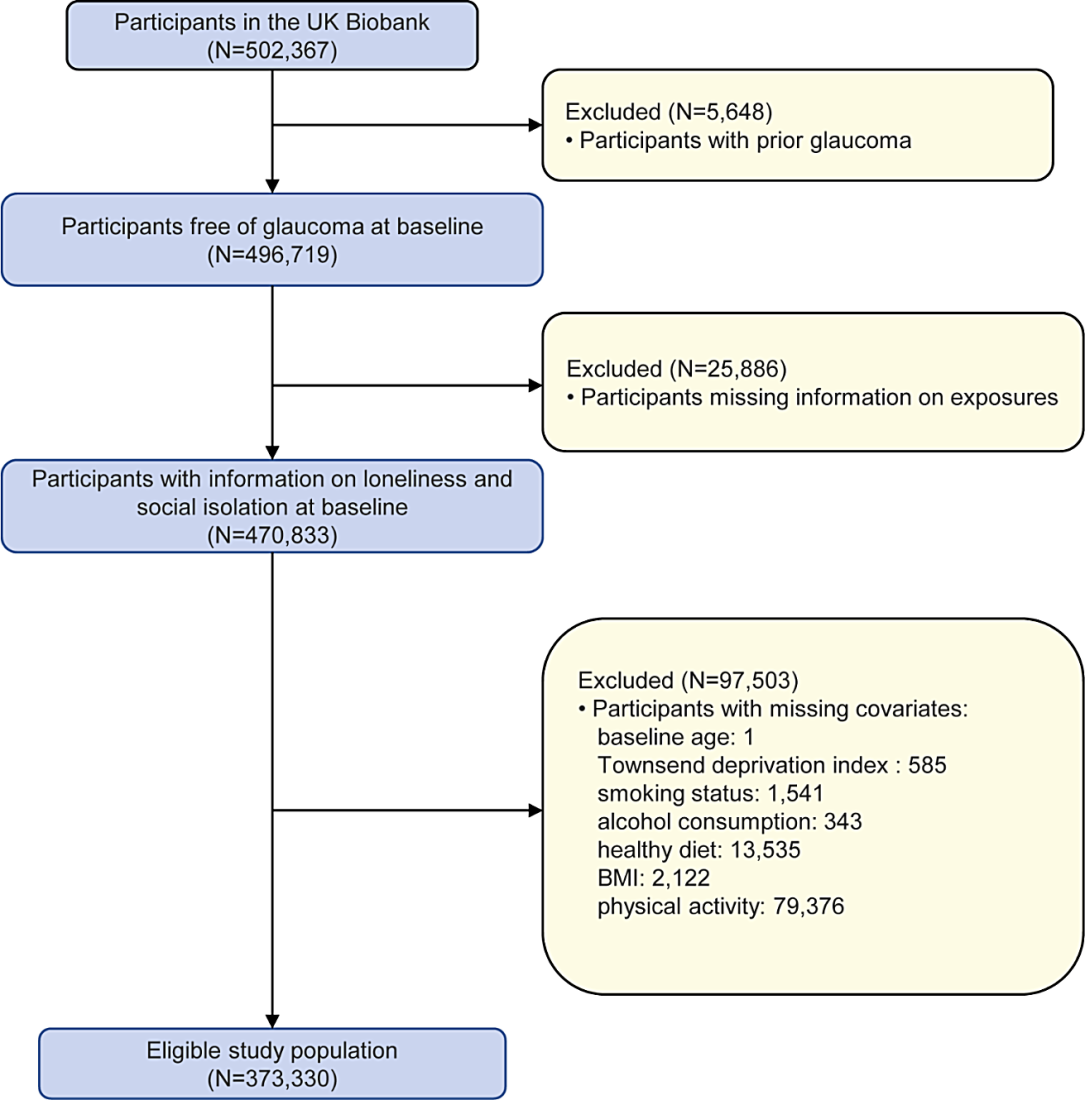
Flowchart of the study population

In this study, participants with prevalent glaucoma at baseline or participants with missing data were excluded. Finally, a total of 373,330 participants were included in our main analysis (Fig. 1).

### Definition of loneliness and social isolation scales

The scales of loneliness and social isolation were derived from self-reported questions at recruitment and were consistent with several previous studies [10, 11]. Loneliness was assessed by two questions derived from the revised UCLA loneliness scale [18]: “Do you often feel lonely?” (1 point for the answer “yes”) and “How often are you able to confide in someone close to you?” (1 point for the answer “never or almost never”). Individuals were defined as lonely if they had a total score of 2. Social isolation was assessed by three questions which were similar to those of the validated Berkman-Syme social network index [19]: (1) “How often do you visit friends or family or have them visit you?” (1 point for less than one visit per month); (2) “Which of the following (sports club or gym, pub or social club, religious group, adult education class, other group activity) do you engage in once a week or more often?” (1 point for participating none of these activities); and (3) “Including yourself, how many people are living together in your household?” (1 point for living alone). Individuals with a total score of 2 or more were categorized as socially isolated.

### Outcomes

In this study, incident cases of glaucoma were ascertained by International Classification of Diseases (ICD) diagnosis codes (10th Revision, ICD-10, or 9th Revision, ICD-9) and participants’ self-reported diagnoses. We ascertained cases through ICD-10 codes H401, H408, and H409; ICD-9 codes 365; self-reported glaucoma (20002: 1277) and answer of glaucoma in a question about eye problems or disorders (data field 6148) and the age when glaucoma was diagnosed (data field 4689). Participants with glaucoma at baseline were excluded from the study. Clinical entities associated with these definitions are listed in Supplementary Table S1. The follow-up time was calculated from the time of recruitment to the time of loss to follow-up, the time of death, the time of diagnosis, or the censoring date (01 May 2022).

### Covariates

We considered the following characteristics as the potential covariates: age at recruitment, sex, race (white, others), education (college or university degree, others), the Townsend deprivation index, smoking status (never, previous, current), moderate drinking, physical activity, healthy diet, body mass index (BMI), history of hypertension, history of hypercholesterolemia, and history of diabetes. The socioeconomic level was represented by the Townsend deprivation index, which was based on the participants’ post code at enrollment [20]. Moderate drinking was set as ≤ 14 g/d for women and ≤ 28 g/d for men [21]. Physical activity was represented by the metabolic equivalent task (MET) minutes per week, which calculated the sum of energy used for walking, moderate, and vigorous activities. A healthy diet was evaluated based on the increased intake of fruits, vegetables, and fish and decreased intake of processed and red meats (Supplementary Table S2). A healthy diet was defined as one that met at least two criteria [22]. History of hypertension was defined as having a systolic blood pressure of 140 mmHg, a diastolic blood pressure of 90 mmHg, a self-reported history of hypertension, or being on antihypertensive medication. Self-reported history of hypercholesterolemia or use of lipid-lowering medications was considered hypercholesterolemia. The definition of diabetes included self-reported diabetes, the use of insulin or diabetic pills, and HbA1c ≥ 6.5% (48 mmol/mol).

### Statistical analysis

Baseline characteristics were presented by the status of loneliness and social isolation. Continuous variables were summarized by mean (SD) and categorical variables were summarized by frequency (%). Baseline variables were compared by the analysis of variance or the Kruskal-Wallis test for continuous data and the Pearson chi-square test for categorical data, as appropriate. Cumulative incidence and incidence rates for glaucoma were calculated per 1000 person-years of follow-up.

COX proportional hazards models were used to explore the association of loneliness and social isolation with the incidence of glaucoma. The proportional hazards assumptions were verified based on Schoenfeld residuals, and no violations were present. The results were presented as hazard ratios (HRs) and 95% confidence intervals (CIs). We created three models, each with an increasing number of confounding variables. Model 1 was adjusted for age and sex. Model 2 was additionally adjusted for race, education level, and Townsend deprivation index. Model 3 was further adjusted for smoking status, moderate drinking consumption, physical activity, healthy diet, BMI, and history of hypertension, hypercholesterolemia, and diabetes. Besides, considering the possibility of reporting bias in self-reported diagnoses, we further assessed the impacts of loneliness and social isolation on ICD codes-defined glaucoma and self-reported glaucoma separately.

Additionally, we conducted subgroup analyses stratified by age (by tertile distribution: ≤52, 53–61, or ≥ 62 years), sex, and race. To lessen the underlying influence of reverse causality, we also conducted a sensitivity analysis by excluding participants who developed glaucoma within a 2-year follow-up.

All analyses were performed using STATA/SE (version 16.0, StataCorp., College Station, TX, USA) and R software (version 4.2.2, R Foundation for Statistical Computing, Vienna, Austria). A two-sided *P* < 0.05 is considered statistically significant.

## Results

### Baseline characteristics

The baseline characteristics of the study population were presented in Table 1. The study sample for the main analysis comprised 373,330 participants (mean age: 56.2 ± 8.1 years, 52.8% female), of whom 51,977 (13.9%) were classified as socially isolated, and 16,909 (4.5%) were classified as being lonely. Participants with social isolation or loneliness were more likely to be male and socioeconomically deprived. Besides, they were more likely to have unhealthy lifestyles, including less physical activity level, smoking, unhealthy diet, and non-moderate alcohol consumption. Additionally, they had higher proportions of self-reported hypertension, hypercholesterolemia, and diabetes.

**Table 1:** Baseline characteristics of participants in the UK Biobank by loneliness and social isolation status

		Loneliness			Social isolation		
Characteristics	Overall (*N* = 373,330)	No (*N* = 356,421)	Yes (*N* = 16,909)	P value	No (*N* = 321,353)	Yes (*N* = 51,977)	P value
Age, years, mean (SD)	56.2 (8.1)	56.3 (8.1)	55.8 (8.0)	< 0.001	56.3 (8.1)	55.8 (7.9)	< 0.001
Female, No. (%)	197,192 (52.8)	189,285 (53.1)	7,907 (46.8)	< 0.001	172,329 (53.6)	24,863 (47.8)	< 0.001
Townsend deprivation index, mean (SD)	-1.4 (3.0)	-1.5 (3.0)	-0.5 (3.4)	< 0.001	-1.6 (2.9)	-0.5 (3.4)	< 0.001
White, No. (%)	356,057 (95.6)	340,157 (95.7)	15,900 (94.4)	< 0.001	307,413 (95.9)	48,644 (94.0)	< 0.001
College or university degree, No. (%)	132,622 (35.5)	128,224 (36.0)	4,398 (26.0)	< 0.001	113,600 (35.4)	19,022 (36.6)	< 0.001
BMI, kg/m^2^, mean (SD)	27.3 (4.7)	27.2 (4.7)	28.3 (5.4)	< 0.001	27.3 (4.6)	27.6 (5.1)	< 0.001
Total physical activity, MET-mins/wk, mean (SD)	2656.1 (2705.3)	2658.2 (2691.6)	2612.7 (2980.5)	0.03	2710.4 (2703.1)	2320.8 (2694.9)	< 0.001
Healthy diet, No. (%)	173,192 (46.4)	166,505 (46.7)	6,687 (39.6)	< 0.001	151,040 (47.0)	22,152 (42.6)	< 0.001
Smoking status, No. (%)				< 0.001			< 0.001
Never	204,786 (54.9)	196,626 (55.2)	8,160 (48.3)		178,330 (55.5)	26,456 (50.9)	
Previous	130,932 (35.1)	125,100 (35.1)	5,832 (34.5)		113,370 (35.3)	17,562 (33.8)	
Current	37,612 (10.1)	34,695 (9.7)	2,917 (17.2)		29,653 (9.2)	7,959 (15.3)	
Moderate drinking, No. (%)	144,396 (38.7)	138,751 (38.9)	5,645 (33.4)	< 0.001	124,895 (38.9)	19,501 (37.5)	< 0.001
Self-reported diseases							
Hypertension	199,492 (53.4)	189,998 (53.3)	9,494 (56.2)	< 0.001	170,998 (53.2)	28,494 (54.8)	< 0.001
Hypercholesterolemia	65.926 (17.7)	62,188 (17.5)	3,738 (22.1)	< 0.001	56,066 (17.5)	9,860 (19.0)	< 0.001
Diabetes	21,448 (5.8)	19,855 (5.6)	1,593 (9.4)	< 0.001	17,428 (5.4)	4,020 (7.7)	< 0.001

*Abbreviations* BMI = body mass index; MET = metabolic equivalent of task

* Continuous variables are described as mean (SD), while categorical variables are displayed as frequency (%)

### Associations of loneliness and social isolation with the incidence of glaucoma

During a median 13.1 years (interquartile range: 12.3–13.9) of follow-up, we recorded 6489 cases of glaucoma after the study baseline. Participants with loneliness or social isolation had higher cumulative incidences of glaucoma (Table 2). The associations of loneliness and social isolation with the risk of glaucoma were presented in Table 3. In the minimally adjusted model (model 1), participants who were classified as loneliness, compared with those without loneliness, were associated with a higher risk of glaucoma (HR: 1.20; 95% CI: 1.07–1.34; *P* = 0.002). Similarly, the HR of social isolation on glaucoma was 1.11 (95% CI: 1.04–1.19) when adjusted for age and sex. After further adjustment for socioeconomic factors, including race, education level, and Townsend deprivation index (model 2), these risks did not change appreciably. In the fully adjusted model (model 3), both loneliness (HR: 1.16; 95% CI: 1.04–1.30; *P* = 0.01) and social isolation (HR: 1.08; 95% CI: 1.01–1.16; *P* = 0.03) were associated with an increased risk of glaucoma. When further exploring the effects of loneliness and social isolation with glaucoma defined by different sources, we found significant associations in glaucoma defined by ICD codes. After full adjustment, the HR of loneliness on ICD codes-defined glaucoma was 1.20 (95% CI: 1.07, 1.34) and the HR of social isolation was 1.08 ((95% CI: 1.01, 1.17). Nevertheless, there were no significant results for self-reported glaucoma.

**Table 2:** Cumulative incidence and incidence rates for primary outcomes

		All glaucoma			ICD defined glaucoma			Self-reported glaucoma		
	No. at risk	Cumulative incidence	No. of events/ Person-years	IR per 1,000 person years (95% CI)	Cumulative incidence	No. of events/ Person-years	IR per 1,000 person years (95% CI)	Cumulative incidence	No. of events/ Person-years	IR per 1,000 person years (95% CI)
**Loneliness**										
no	356,421	1.73	6160/4,554,166	1.35 (1.32, 1.39)	1.58	5554/4,554,166	1.22 (1.19, 1.25)	0.17	606/4,554,166	0.13 (0.12, 0.14)
yes	16,909	1.95	329/214,018	1.54 (1.38, 1.71)	1.82	308/214,018	1.44 (1.29, 1.61)	0.12	21/214,018	0.10 (0.06, 0.15)
**Social isolation**										
no	321,353	1.73	5570/4,429,902	1.35 (1.32, 1.39)	1.56	5028/4,114,054	1.22 (1.19, 1.26)	0.17	542/4,114,054	0.13 (0.12, 0.14)
yes	51,977	1.77	919/703,494	1.40 (1.32, 1.50)	1.60	834/654,130	1.27 (1.19, 1.36)	0.16	85/654,130	0.13 (0.11, 0.16)

*Abbreviations* CI = confidence interval; IR = incidence rate

**Table 3:** Associations of loneliness and social isolation with the incidence of glaucoma

	All glaucoma		ICD defined glaucoma		Self-reported glaucoma	
	HR (95%CI)	P value	HR (95%CI)	P value	HR (95%CI)	P value
**Loneliness**						
Model 1	1.20 (1.07, 1.34)	0.002	1.25 (1.11, 1.40)	< 0.001	0.74 (0.48, 1.14)	0.173
Model 2	1.17 (1.04, 1.30)	0.007	1.21 (1.07, 1.35)	0.001	0.79 (0.51, 1.22)	0.287
Model 3	1.16 (1.04, 1.30)	0.009	1.20 (1.07, 1.34)	0.002	0.81 (0.53, 1.26)	0.355
**Social isolation**						
Model 1	1.11 (1.04, 1.19)	0.002	1.13 (1.05, 1.21)	0.001	0.99 (0.79, 1.26)	0.993
Model 2	1.09 (1.01, 1.17)	0.020	1.09 (1.02, 1.18)	0.018	1.03 (0.81, 1.29)	0.828
Model 3	1.08 (1.01, 1.16)	0.033	1.08 (1.01, 1.17)	0.033	1.04 (0.83, 1.31)	0.733

* Model 1 was adjusted for age (continuous) and sex (female, male). Model 2 was adjusted for model 1 + race (white, others), education (college or university degree, others), and Townsend deprivation index (continuous). Model 3 was adjusted for model 2 + smoking status (never, past, current), moderate drinking (yes, no), healthy diet (yes, no), total physical activity level (MET-minutes /week, continuous), BMI, self-reported history of hypertension (yes, no), self-reported history of hypercholesterolemia (yes, no), and self-reported history of diabetes (yes, no)

Multiplicative interaction analysis further revealed that the association between loneliness and the incidence of glaucoma was not modified by age, sex, or race (Supplementary Table S3). Besides, the relationship between social isolation and the incidence of glaucoma was unaffected by age and sex. There was an interaction of race on the association between social isolation and glaucoma (p for interaction = 0.02). Considering different age, sex, and race groups may have different intensity levels, subgroup analyses with full adjustments were still conducted. And these associations were not appreciably changed in the stratified analyses (Supplementary Table S4). The major results remained stable in the sensitivity analyses when excluding glaucoma cases that occurred within the first 2 years of follow-up (Supplementary Table S5).

## Discussion

In this large prospective cohort study of the UK Biobank data, we mainly discovered that both loneliness and social isolation were associated with an increased risk of glaucoma. The associations were independent of age, sex, race, socioeconomic status, lifestyle factors, and history of hypertension, hypercholesterolemia, and diabetes. The robustness of these results was demonstrated by stratified and sensitivity analyses.

Glaucoma is a chronic, progressive disease that is among the leading causes of visual impairment all over the world [1, 23]. Upon a glaucoma diagnosis, patients often fear blindness and are plagued with unpleasant emotions, which may consequently evolve into some mental problems [24]. On the other side, emerging evidence has suggested that psychological factors play a crucial role on the development of glaucoma. For instance, glaucoma suspects with anxiety and depression were associated with an increased risk of glaucoma [25]. As an aspect of psychological factors, loneliness represents a source of mental stress and was associated with cardiovascular health [26]. However, little is known about its effect on ocular health.

Many studies have demonstrated that social factors are associated with the risk of glaucoma. For instance, as an indicator of social status, both area and individual level deprivation were associated with late glaucoma manifestation [27]. Recently, a cross-sectional study reported that personal poverty, defined as not driving a personal car to the appointment, and neighborhood-level poverty were both associated with a higher incidence of glaucoma or suspected glaucoma [28]. Additionally, it was suggested that many socioeconomic factors, such as smoking, not having a car for transportation to eye exams, and living alone, have been associated with a lack of follow-up after glaucoma screening clinics [29]. Hence, early identification of these risk factors may help improve compliance. Social isolation, as an element of social characteristics, has emerged as a public health issue [30, 31]. Our findings help complement the broad spectrum of published reports on the association of social isolation with health-related outcomes.

We speculate several potential mechanisms to explain our findings. First, it has been suggested that loneliness and social isolation enhance stress reactivity, which is associated with activation of the hypothalamic-pituitary-adrenal axis [13, 14, 32]. Increased metabolic stress has been reported to contribute to the damage and dysfunction of the glaucomatous neurovascular unit [33], thus accelerating the progression of glaucoma. Second, elevated levels of glucocorticoids (GCs), which are regulated by the hypothalamic-pituitary-adrenal axis, have side effects on glaucoma by inducing morphological changes in the trabecular meshwork and leading to elevated IOP [34]. Third, loneliness and social isolation increase the levels of inflammation and oxidative stress by upregulating sympathetic activity [35]. Activation of inflammasome is reported to accelerate RGC degeneration and neuroinflammation in glaucomatous pathology [15]. Besides, oxidative stress is associated with mitochondrial dysfunction in glaucomatous RGC degeneration and serves as a key factor in RGC death [16]. All these stimuli are involved in the mediation of glaucomatous injury. Fourth, social disconnection may restrict older adults from medical support and healthcare resources [36], ultimately leading to insufficiently timely diagnosis or treatment of glaucoma.

The strengths of this study include its prospective study design, large sample size, long-term follow-up, and comprehensive definitions of covariates and outcomes. As far as we are aware, our current study is the first to report that both loneliness and social isolation are associated with a higher risk of glaucoma, independent of socioeconomic factors, lifestyle factors, and health-related factors.

However, this study had several limitations. First, the measurements of loneliness and social isolation in the UK Biobank were self-reported and had not been formally verified. However, these questions were adapted from validated scales [18, 19], and widely adopted in previous studies [9–12]. Second, we used ICD codes to identify cases that required surgery or hospitalization for the condition and therefore may have missed cases diagnosed only on an outpatient basis. Therefore, we used self-reported information to capture these cases. However, a limitation of self-reported data is the susceptibility to recall errors, particularly concerning misinterpretation of the disease or age at diagnosis. This may partially explain why our findings were not significant when restricted to self-reported glaucoma. Third, due to the lack of clinical glaucoma screening, there may be a subset of participants with clinically undiagnosed glaucoma, which could lead to misclassification bias. Similar to other large population-based studies, our present study failed to specify the association between loneliness and social isolation with different glaucoma subtypes due to the lack of phenotypic information in the UK Biobank. Distinguishing between these subtypes of glaucoma with different pathophysiologic mechanisms may help to better understand the effects of loneliness and social isolation on glaucoma. Fourth, the measurements of loneliness, social isolation, and other covariates were evaluated at baseline, which may diminish their relevance if measured too far from the glaucoma diagnosis. However, the fact that previous studies have employed this method explains in part the persuasiveness of this approach and the reliability of our results. Fifth, because we used non-randomized observational data, we were unable to conclude causality, and thus a major concern of this study was reverse causation. Nevertheless, our results remained robust in the sensitivity analysis, suggesting that the observed associations were less likely to be confounded by reverse causation. In addition, though we had carefully considered the adjustments of potential confounders, the possibility of residual confounding cannot be ruled out. Finally, the UK Biobank cohort is not representative of the characteristics of the entire UK population. However, it has been argued that the exposure-disease relationship in the UK Biobank may have broad reliability [37].

## Conclusions

In conclusion, the present finding suggests that both loneliness and social isolation are associated with an increased risk of glaucoma. Our finding underscores the necessity of positive mental and social network intervention for individuals at high risk of glaucoma. Further research is needed to clarify the effects of loneliness and social isolation on specific subtypes of glaucoma.

### Electronic supplementary material

Below is the link to the electronic supplementary material.

Supplementary Material 1

## Data Availability

Bona fide researchers can register and apply to use the UK Biobank dataset at http://ukbiobank.ac.uk/register-apply/.
