# Supplementary material for: Loneliness and social isolation are associated with an increased risk of glaucoma: a UK Biobank cohort study
Source: BMC Public Health. 2024 Aug 5;24:2109. doi: 10.1186/s12889-024-19649-6 (PMC11299252; doi:10.1186/s12889-024-19649-6)
Supplement: Supplementary file 1 — Supplementary Material 1 [file 12889_2024_19649_MOESM1_ESM.docx]

**SUPPLEMENTARY MATERIALS**

**Loneliness and social isolation are associated with an increased risk of glaucoma: a UK Biobank cohort study**

**Contents**

**Supplementary Table S1.** Definitions and codes used in ascertainment of glaucoma and number of cases identified

**Supplementary Table S2.** Definitions of a healthy diet.

**Supplementary Table S3.** P values on interaction of loneliness, social isolation and stratification variables with glaucoma

**Supplementary Table S4.** Associations of loneliness and social isolation with the incidence of glaucoma stratified by age, gender, and race

**Supplementary Table S5.** Associations of loneliness and social isolation with the incidence of glaucoma after excluding outcomes occurred within 2 years of follow-up

**Supplementary Table S1.** Definitions and codes used in ascertainment of glaucoma and number of cases identified

|  | **Glaucoma** | **Number of cases** |
| --- | --- | --- |
| **ICD** | ICD-9  365: Glaucoma | 5862 |
|  | ICD-10  H401: Primary open-angle glaucoma  H408: Other glaucoma  H409: Glaucoma, unspecified |  |
| **Self-reported** | 20002  1277: Glaucoma  6148: Eye problems/disorders  4689: Age glaucoma diagnosed | 627 |

**Supplementary Table S2.** Definition of a healthy diet.

| **Component** | **Questions from the UK Biobank** | **Criteria** |
| --- | --- | --- |
| Total fruit and vegetable intake | About how many pieces of FRESH fruit would you eat per DAY? (Count one apple, one banana, 10 grapes etc as one piece; put '0' if you do not eat any)  On average how many heaped tablespoons of COOKED vegetables would you eat per DAY? (Do not include potatoes; put '0' if you do not eat any)  On average how many heaped tablespoons of SALAD or RAW vegetables would you eat per DAY? (Include lettuce, tomato in sandwiches; put '0' if you do not eat any) | > 4.5 pieces or servings a week (3 Tablespoons of vegetables = one serving) |
| Total fish intake | How often do you eat oily fish? (e.g. sardines, salmon, mackerel, herring)  How often do you eat other types of fish? (e.g. cod, tinned tuna, haddock) | > 2 per week |
| Processed and red meat intake | How often do you eat processed meats (such as bacon, ham, sausages, meat pies, kebabs, burgers, chicken nuggets)?  How often do you eat beef? (Do not count processed meats)  How often do you eat lamb/mutton? (Do not count processed meats) | processed meat: <=2 per week & red meat: <=5 per week |
| A healthy diet was defined as “yes” if at least two of the criteria were met, otherwise was defined as “no”. | | |

**Supplementary Table S3.** P values on interaction of loneliness, social isolation and stratification variables with glaucoma

|  |  | **Glaucoma** | | |
| --- | --- | --- | --- | --- |
|  |  | Age | Sex | Race |
| **Loneliness** | No | [reference] | | |
|  | Yes | 0.22 | 0.18 | 0.08 |
| **Social isolation** | No | [reference] | | |
|  | Yes | 0.16 | 0.29 | 0.02 |

* *Models were adjusted for age (continuous), sex (female, male), race (White people, non‐White people [including Black people, Asian people, and multiethnic participants]), Townsend deprivation index (continuous), education (college or university degree, others), smoking status (never, past, current), moderate drinking (yes, no), healthy diet (yes, no), total physical activity level (MET-minutes /week, continuous), BMI, self-reported history of hypertension (yes, no), self-reported history of hypercholesterolemia (yes, no), and self-reported history of diabetes (yes, no). The strata variable was not included in the model when stratifying by itself.

Abbreviation: BMI = body mass index.

**Supplementary Table S4.** Associations of loneliness and social isolation with the incidence of glaucoma stratified by age, gender, and race

|  |  | **Loneliness** | | | **Social isolation** | | |
| --- | --- | --- | --- | --- | --- | --- | --- |
|  | N | Cases/person-years | HR (95% CI) | P value | Cases/person-years | HR (95% CI) | P value |
| **Age** |  |  |  |  |  |  |  |
| <=52 | 125,609 | 50/78830 | 1.25 (0.93, 1.68) | 0.13 | 114/236986 | 1.00 (0.82, 1.22) | 0.99 |
| >=53 <=61 | 128,946 | 124/76734 | 1.26 (1.05, 1.52) | 0.01 | 330/242827 | 1.07 (0.95, 1.21) | 0.24 |
| >=62 <=73 | 118,775 | 155/58454 | 1.06 (0.90, 1.24) | 0.49 | 475/174317 | 1.10 (0.99, 1.21) | 0.06 |
| **Sex** |  |  |  |  |  |  |  |
| Male | 176,138 | 165/112710 | 1.09 (0.93, 1.27) | 0.30 | 514/337415 | 1.12 (1.02, 1.24) | 0.02 |
| Female | 197,192 | 164/101308 | 1.25 (1.06, 1.46) | 0.01 | 405/316715 | 1.03 (0.92, 1.14) | 0.65 |
| **Race** |  |  |  |  |  |  |  |
| White | 356,057 | 311/201318 | 1.20 (1.07, 1.35) | 0.002 | 831/612681 | 1.06 (0.99, 1.14) | 0.11 |
| Others | 16,304 | 15/11844 | 0.72 (0.43, 1.22) | 0.22 | 82/38667 | 1.40 (1.09, 1.80) | 0.01 |

*****HR was adjusted for age (continuous), sex (female, male), race (White people, non‐White people [including Black people, Asian people, and multiethnic participants]), Townsend deprivation index (continuous), education (college or university degree, others), smoking status (never, past, current), moderate drinking (yes, no), healthy diet (yes, no), total physical activity level (MET-minutes /week, continuous), BMI, self-reported history of hypertension (yes, no), self-reported history of hypercholesterolemia (yes, no), and self-reported history of diabetes (yes, no). The strata variable was not included in the model when stratifying by itself.

Abbreviations: BMI = body mass index; CI = confidence intervals; HR = hazard ratio.

**Supplementary Table S5.** Associations of loneliness and social isolation with the incidence of glaucoma after excluding outcomes occurred within 2 years of follow-up

|  | **All glaucoma** | | **ICD defined glaucoma** | | **Self-reported glaucoma** | |
| --- | --- | --- | --- | --- | --- | --- |
|  | HR (95%CI) | P value | HR (95%CI) | P value | HR (95%CI) | P value |
| **Loneliness** | | | | | | |
| Model 1 | 1.17 (1.04, 1.31) | 0.008 | 1.23 (1.09, 1.38) | 0.001 | 0.58 (0.34, 0.98) | 0.043 |
| Model 2 | 1.14 (1.01, 1.28) | 0.030 | 1.19 (1.05, 1.34) | 0.005 | 0.62 (0.36, 1.06) | 0.078 |
| Model 3 | 1.13 (1.01, 1.27) | 0.036 | 1.18 (1.04, 1.33) | 0.007 | 0.64 (0.38, 1.09) | 0.101 |
| **Social isolation** | | | | | | |
| Model 1 | 1.12 (1.05, 1.21) | 0.002 | 1.13 (1.05, 1.22) | 0.001 | 1.02 (0.79, 1.31) | 0.871 |
| Model 2 | 1.09 (1.02, 1.18) | 0.015 | 1.10 (1.02, 1.19) | 0.015 | 1.05 (0.82, 1.34) | 0.723 |
| Model 3 | 1.09 (1.01, 1.17) | 0.024 | 1.09 (1.01, 1.18) | 0.027 | 1.06 (0.83, 1.36) | 0.635 |

*Model 1 was adjusted for age (continuous) and sex (female, male). Model 2 was adjusted for model 1 + race (white, others), education (college or university degree, others), and Townsend deprivation index (continuous). Model 3 was adjusted for model 2 + smoking status (never, past, current), moderate drinking (yes, no), healthy diet (yes, no), total physical activity level (MET-minutes /week, continuous), BMI, self-reported history of hypertension (yes, no), self-reported history of hypercholesterolemia (yes, no), and self-reported history of diabetes (yes, no).

Abbreviations: BMI = body mass index; CI = confidence intervals; HR = hazard ratio.
